# Dual-targeting strategy enables extremely potent and broad inhibition of emerging MERS-related coronaviruses

**DOI:** 10.1038/s41421-025-00827-8

**Published:** 2025-08-26

**Authors:** Fanke Jiao, Suya Jin, Qian Wang, Wei Xu, Xinling Wang, Fei Sun, Lu Lu, Shibo Jiang, Yun Zhu, Shuai Xia

**Affiliations:** 1 Shanghai Public Health Clinical Center, Key Laboratory of Medical Molecular Virology (MOE/NHC/CAMS), Shanghai Institute of Infectious Disease and Biosecurity, School of Basic Medical Sciences, Shanghai Frontiers Science Center of Pathogenic Microbes and Infection, Fudan University, Shanghai, China; 2 National Laboratory of Biomacromolecules, Institute of Biophysics, Chinese Academy of Sciences, Beijing, China

**Keywords:** Cell biology, Structural biology

Dear Editor,

Recently, the receptors of several MERS-related coronaviruses (MERSr-CoVs), including MjHKU4r-CoV-1, NeoCoV, PDF-2180, MOW15-22, PnNL2018B, HKU5-CoV-1 and HKU5-CoV-2 (Fig. 1a), have been continuously identified^1–4^. Compared with the prototypical MERS-CoV, these MERSr-CoVs exhibit substantial alterations in their spike (S) proteins, particularly within the receptor-binding domain (RBD) (Fig. 1b; Supplementary Fig. S1a, b). Remarkably, many MERSr-CoVs diverge from the canonical use of DPP4 as a receptor^4^ (Fig. 1a), a hallmark of Merbecoviruses, and instead show the capacity to utilize ACE2 for cell entry^1–3^. For instance, two bat-derived MERSr-CoVs, NeoCoV and PDF-2180^2,5^, have been shown to use bat ACE2 as their entry receptor and display broad non-bat mammal ACE2 recognition^2^, thus potentially facilitating their cross-species transmission to humans in the future. Importantly, the receptor-binding modes of these viruses are structurally distinct from that of MERS-CoV or SARS-CoV-2, allowing them to substantially evade immune responses induced by previously developed vaccines^6^. These findings highlight the urgent need to develop antiviral agents capable of counteracting a wide range of emerging MERSr-CoVs^6^.

**Fig. 1:**
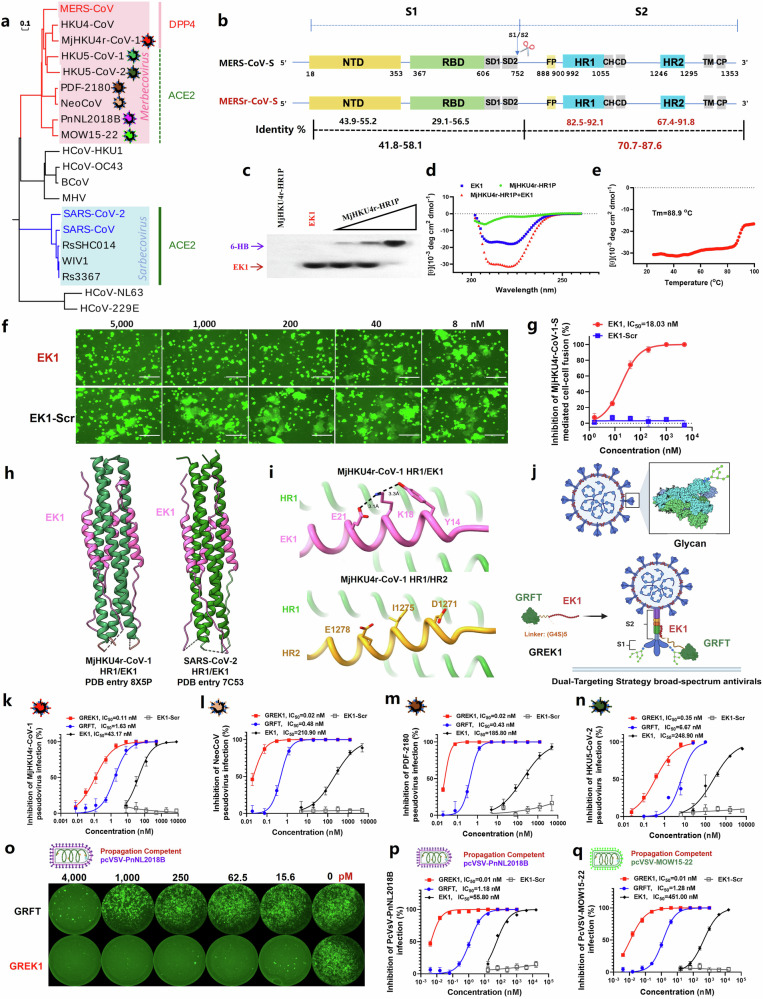
GREK1 potently inhibited infection of multiple MERSr-CoVs. **a** Neighbor-joining (NJ) tree created with the S sequences of representative CoVs from α and β coronaviruses. **b** The identity of function domains in S protein of MERSr-CoVs, compared with those of MERS-CoV. **c** Native polyacrylamide gel electrophoresis (N-PAGE) analysis of the MjHKU4r-HR1P/EK1 interaction. New 6-HB bands are marked with a purple arrow, while the red arrow indicates the EK1 band. **d** Circular dichroism (CD) spectra of MjHKU4r-HR1P, EK1 and MjHKU4r-HR1P/EK1 complex. **e** Melting curves of the complexes formed by MjHKU4r-HR1P and EK1. **f** Representative images of cell–cell fusion between effector cells expressing MjHKU4r-S and target cells (Caco-2) after coculture for 2 h in the presence of EK1 or EK1-Scrambled (EK1-Scr) with indicated concentrations. Scale bars, 150 μm. **g** The broad-spectrum and potency of EK1 against cell–cell fusion mediated by MjHKU4r-S. **h** Cartoon representation of the crystal structures of MjHKU4r-CoV-1 HR1/EK1 complex (left, PDB: 8X5P) and SARS-CoV-2-HR1/EK1 complex (right, PDB: 7C53). HR1 and EK1 are colored green and hot pink, respectively. **i** Structural comparison between MjHKU4r-CoV-1 HR1/HR2 (PDB: 8X5O) and HR1/EK1. Important residues are highlighted as sticks in the zoom-in view. **j** Schematic diagram of the bivalent fusion inhibitor GREK1, comprising GRFT, EK1 and an intervening (G4S)5 linker targeting either the glycans in S1 subunit or HR1 in S2 subunit of S protein of MERSr-CoVs. **k**–**n** The efficacy of GREK1 against pseudotyped MjHKU4r-CoV-1 (**k**), NeoCoV (**l**), PDF-2180 (**m**) and HKU5-CoV-2 (**n**) infection. **o**–**q** Representative images of GRFT and GREK1 inhibiting propagation-competent pcVSV-PnNL2180B infection (**o**). Quantification of the antiviral potency of GRFT and GREK1 against pcVSV-PnNL2180B (**p**) or pcVSV-MOW15-22 (**q**) infection.

Here, we found that S2 subunits of these MERSr-CoVs exhibited high conservation, especially in their HR1 regions, a critical functional domain involved in viral 6-HB fusion core formation, all of which showed greater than 80% sequence identity with MERS-CoV-HR1 (Fig. 1b; Supplementary Fig. S1b, c), suggesting that their HR1 regions might serve as a potential target site. Consistently, all HR1-derived peptides (HR1Ps) of these MERSr-CoVs can interact with the pan-CoV fusion inhibitor EK1^7^, thus forming new bands in the native polyacrylamide gel electrophoresis (N-PAGE) (Fig. 1c; Supplementary Fig. S2).

Meanwhile, MjHKU4r-CoV-1 HR1P was used as the representative MERSr-CoV HR1-derived peptide, which exhibited a predominantly random coil structure with low α-helicity in the circular dichroism (CD) spectrum (Fig. 1d). Notably, the mixture of HR1P and EK1 formed a complex with significantly increased α-helicity. Thermal denaturation analysis further demonstrated that the HR1P/EK1 complex had high thermostability, with a melting temperature (Tm) of 88.9 °C (Fig. 1e). These findings confirm that EK1 can effectively and competitively bind to the HR1 region of MERSr-CoVs, resulting in the formation of a stable heterologous 6-HB.

Importantly, EK1 significantly inhibited MjHKU4r-CoV-1 S-mediated membrane fusion in a dose-dependent manner, with an IC_50_ of 18.03 nM (Fig. 1f, g), approximately 10-fold more potent than its inhibition of prototype MERS-CoV S-mediated fusion^7^. Furthermore, EK1 also potently blocked NeoCoV, PDF-2180, MOW15-22 and PnNL2018B S-mediated cell–cell fusion, with IC_50_ values ranging from 51.98 nM to 86.39 nM (Supplementary Fig. S3). These results collectively demonstrate that EK1 exerts broad and potent fusion-inhibitory activity against these MERSr-CoVs.

Meanwhile, we successfully resolved the crystal structure of the MjHKU4r-HR1/EK1 complex (PDB: 8X5P), whose overall architecture closely resembles that of the SARS-CoV-2-HR1/EK1 complex (PDB: 7C53)^8^ (Fig. 1h; Supplementary Fig. S4a, b). The structure revealed that EK1 specifically targets the MjHKU4r-HR1 domain (Fig. 1h, i; Supplementary Table S1), competitively displacing the viral HR2 region to form a stable pseudo-6-HB, thereby effectively blocking the native 6-HB formation that is essential for membrane fusion and viral entry. Sequence alignment showed that EK1 shares a high homology with the HR2 domain of MjHKU4r-CoV-1, contributing to its strong binding affinity for the HR1 region (Fig. 1i; Supplementary Fig. S4a). Notably, EK1 also demonstrated enhanced structural stability compared to the native viral HR2 peptide. Structural analysis further indicated that in EK1, the residue Lys18 forms salt bridges with Glu21 and Tyr14, contributing to the solubility and integrity of its helical structure (Fig. 1i). In contrast, in the MjHKU4r-CoV-1 HR2 domain, Ile1275 occupies a comparable position between Asp1271 and Glu1278, disrupting potential interactions and reducing structural stability (Fig. 1i).

Additionally, we used Alphaold3^9^ to predict the structures of EK1 bound to the HR1 regions of these viruses. Comparison of these predicted structures with our resolved crystal structure (Supplementary Fig. S4c) showed that EK1 can bind the HR1 trimer of all these MERSr-CoVs in a highly conserved conformation, aligning with the high degree of sequence conservation in these viral HR1 regions. According to the sequence alignment, these MERSr-CoVs exhibit limited point mutations in HR1. A detailed analysis of these mutation sites revealed that none of them interferes with or weakens EK1’s ability to bind HR1. For example, when L1001 in MjHKU4r-CoV-1 is mutated to I in NeoCoV, it maintains a hydrophobic interaction with I31 of EK1; however, when mutated to Q in PnNL2018B, it tends to interact with E35 of EK1. This demonstrates that EK1 possesses the flexibility to match HR1 by altering the side-chain conformations of its own residues, thereby displaying broad-spectrum antiviral activity (Supplementary Fig. S4).

Given the high degree of glycosylation on the surface of coronaviruses, viral glycans represent a second conserved target for antiviral intervention. Griffithsin (GRFT), a well-characterized antiviral lectin, can specifically bind to these glycans on the S protein^10^, thereby inhibiting viral entry. Building on this principle, we further evaluated the fusion-inhibitory activity of a bivalent fusion inhibitor, GREK1, which combines the glycan-targeting capability of GRFT with HR1-binding domain of EK1 (Fig. 1j).

As anticipated, GREK1 potently inhibited MjHKU4r-CoV-1, NeoCoV, PDF-2180, MOW15-22 and PnNL2018B S-mediated cell–cell fusion in a dose-dependent manner, with IC_50_ values of 0.04–0.27 nM (Supplementary Fig. S5). These IC_50_ values represent 12- to 24-fold greater potency than GRFT alone (IC_50_: 0.79–4.29 nM) and 141- to 1415-fold greater potency than EK1 alone (IC_50_: 22.53–83.29 nM) (Supplementary Fig. S5). Additionally, the S protein of HKU5-CoV-2 also drove significant membrane fusion on the Caco-2-*Pipistrellus nathusii* (*P.nat)*-ACE2 cells (Supplementary Fig. S6a), which could be completely inhibited by EK1 (5 µM). Further, GREK1 showed potent fusion-inhibitory activity against HKU5-CoV-2 with IC_50_ of 0.19 nM, significantly exceeding GRFT alone (IC_50_: 3.90 nM) or EK1 alone (IC_50_: 223.00 nM) (Supplementary Fig. S6b).

To further evaluate the specific antiviral activity of GREK1, we tested its efficacy in blocking infection using an HIV-based pseudovirus (PsV) system^11,12^. Strikingly, GREK1 demonstrated significantly enhanced potency against all tested pseudotyped MjHKU4r-CoV-1, NeoCoV, PDF-2180 and HKU5-CoV-2, with IC_50_ values of 0.02–0.35 nM (Fig. 1k–n). These values were markedly lower than those of GRFT (IC_50_: 0.43–6.67 nM) and EK1 (IC_50_: 43.17–248.90 nM), further supporting the superior antiviral efficacy of the bivalent GREK1 molecule. Furthermore, in propagation-competent recombinant stomatitis virus bearing PnNL2180B-S protein (pcVSV-PnNL2180B) or MOW15-22-S protein (pcVSV-MOW15-22), GREK1 completely suppressed viral replication at low concentrations (Fig. 1o–q; Supplementary Fig. S7). Quantitative analysis revealed that GREK1 exhibited exceptional antiviral potency, with IC_50_ value of 0.01 nM against both pcVSV-PnNL2180B (Fig. 1o, p) and pcVSV-MOW15-22 (Fig. 1q; Supplementary Fig. S7) infection^3^. These values represent about 118–128-fold greater efficacy than GRFT and 5580–45,100-fold greater than EK1 in the same models (Fig. 1o–q; Supplementary Fig. S7). Together, these findings demonstrate that GREK1 is a highly effective antiviral agent with broad-spectrum activity against diverse MERSr-CoVs, greatly surpassing the efficacy of its parental components.

Overall, in the current study, we found that the bivalent recombinant protein GREK1 can simultaneously target both surface glycans and the HR1 region in S protein of various MERSr-CoVs. As a result, GREK1 exhibited exceptionally potent antiviral activity, with IC_50_ values at the low picomolar level, significantly outperforming previously reported antiviral agents^1–4^. During the SARS-CoV-2 pandemic, numerous antiviral agents have been approved for clinical use^13^; however, their efficacy against MERSr-CoVs remains largely unexplored. Particularly, RBD-directed neutralizing antibodies are vulnerable to escape mutations, whereas GREK1 targets two highly conserved domains (viral HR1 and surface glycans), maintaining potent activity against all tested MERSr-CoVs and their potential variants^6^. Moreover, GREK1 exhibits broad-spectrum activity against multiple human coronaviruses, including SARS-CoV-2, SARS-CoV, HCoV-OC43, HCoV-229E, and HCoV-NL63^14^. Therefore, GREK1 possesses strong potential for development as a clinical antiviral agent to combat current and emerging MERSr-CoV or other human coronavirus infections.

## Supplementary information

Supplementary Information
