# Supplementary material for: Dual-targeting strategy enables extremely potent and broad inhibition of emerging MERS-related coronaviruses
Source: Cell Discov. 2025 Aug 26;11:70. doi: 10.1038/s41421-025-00827-8 (PMC12378456; doi:10.1038/s41421-025-00827-8)
Supplement: Supplementary file 1 — Supplementary Information [file 41421_2025_827_MOESM1_ESM.pdf]

Supplementary Information for

Dual-Targeting Strategy Enables Extremely Potent and Broad Inhibition of Emerging MERS-Related Coronaviruses

Fanke Jiao[1†], Suya Jin[1†], Qian Wang[1], Wei Xu[1], Xinling Wang[1], Fei Sun[2], Lu Lu[1*], Shibo Jiang[1*], Yun Zhu[2*], Shuai Xia[1*]

Affiliations:
[1] Shanghai Public Health Clinical Center, Key Laboratory of Medical Molecular Virology (MOE/NHC/CAMS), Shanghai Institute of Infectious Disease and Biosecurity, School of Basic Medical Sciences, Shanghai Frontiers Science Center of Pathogenic Microbes and Infection, Fudan University, Shanghai, China.
[2] National Laboratory of Biomacromolecules, Institute of Biophysics, Chinese Academy of Sciences, Beijing, China.
†These authors contributed equally to this work: Fanke Jiao, Suya Jin
*Corresponding authors: Shuai Xia, Yun Zhu, Shibo Jiang, Lu Lu,

Email: sxia15@fudan.edu.cn (S.X.); zhuyun@ibp.ac.cn (Y.Z.); shibojiang@fudan.edu.cn (S.J.); lul@fudan.edu.cn (L.L.).

This PDF file includes:

Materials and Methods

References

Supplementary figures and tables

Materials and Methods

Cells, viruses, and peptides

293T, Caco2, Hela were obtained from the American Type Culture Collection (ATCC), Caco2-*Pipistrellus nathusii (P.nat)*-ACE2[1] was provided by Professor Huan Yan in Wuhan Institute of Virology. All these cells were cultured with DMEM containing 10% fetal bovine serum. MjHKU4r-CoV-1 was isolated by the Wuhan Institute of Virology. The propagation-competent VSV-S[1] (pcVSV-MOW15-22 and pcVSV-PnNL-2180B) were kindly gifted by Professor Huan Yan. EK1 (SLDQINVTFLDLEYEMKKLEEAIKKLEESYIDLKEL) and other peptides, including EK1-scrambled peptide (EK1-Scr), used in this study were synthesized by Synpeptide Co., Ltd. with ≥95% purity. EK1-Scr sequence was previously described [2].

Plasmids

Plasmid expressing the gene of MjHKU4r-S was a gift from Professor Peng Zhou, and the genes of NeoCoV-S, PDF-2180-S, MOW15-22-S and PnNL2180B-S were a gift from Professor Huan Yan. HKU5-CoV-2-S gene, PC-DNA3.1, pAAV-IRES-EGFP and the luciferase reporter vector (pNL4-3.Luc.R-E-) were maintained in our laboratory.

Cell-cell fusion experimental system and the fusion-inhibitory activity evaluation assay.

This study utilized Caco-2 cells, Caco2-*P.nat*-ACE2 cells, Hela-*Pipistrellus pipistrellus (P.pip)*-ACE2 cells as target cells. Effector cells consisted of 293T cells transfected with plasmids expressing different spike (S) proteins, including MjHKU4r-S, NeoCoV-S, PDF-2180-S, MOW15-22-S and PnNL-2180B-S, designated as 293T/MjHKU4r-S/GFP, 293T/ NeoCoV-S/GFP, 293T/PDF-2180-S/ GFP, 293T/MOW15-22-S/GFP, 293T/PnNL-2180B-S /GFP, and 293T/EGFP,

respectively. Effector cells and target cells were co-cultured in DMEM with 40 μg/mL trypsin for a specified duration. Following incubation, fused and unfused cells were counted using an inverted fluorescence microscope to assess the extent of fusion.

The inhibitory effect of inhibitors on S protein-mediated cell-cell fusion was evaluated using the following method[3]: First, target cells (2 × 10^4 cells/well) were seeded in a 96 well plate 24 hours in advance. Subsequently, effector cells (293T/S/GFP) were added, either with or without the test peptide at indicated concentrations, and co-cultured for 4 hours at 37°C. 293T/EGFP cells transfected with the empty vector served as a negative control. Finally, the fusion rate was calculated based on the counts of fused and unfused cells.

The production, purification and identification of GRFT and GREK1

Optimized gene sequences encoding GRFT and GREK1 with E. coli codon preference were constructed into pET-28a vector and pET-32a vector containing a His6-TRX tag upstream of the multiple cloning sites, respectively. Recombinant plasmids were transformed into E. coli BL21. The transformed BL21 was cultured in the presence of kanamycin (pET-28a) and ampicillin (pET-32a) (50 μg/ml) at 37℃. Pick a single colony and incubate at 37°C with shaking (220 rpm) for 10 h. Until the culture density reached 0.5 at OD_{600}, which was placed on ice for 15 min and then incubated in the presence of 1 mM IPTG at 16℃ with shaking (180 rpm) for 20 h. The cells were harvested at 8,000 rpm for 3 min. The recombinant proteins were purified with Ni–NTA column. Additionally, the TRX tag of GREK1 was removed by TEV Protease (1:100 w/w) cleavage.

Inhibition of pseudotyped MERSr-CoVs infection

To package these MERSr-CoVs pseudoviruses (PsVs), 293T cells were co-transfected with plasmids: pcDNA3.1-S and pNL4–3.luc.RE (HIV-1 backbone bearing the luciferase reporter) by using VigoFect[2]. After 12 h, replace with fresh medium. After additional 48 h, the supernatant containing pseudotyped particles was harvested, then centrifuged at 3000 × g for 10 min, and stored in −80 °C. To detect the inhibitory activity of inhibitors against these MERSr-CoVs PsV infection, used Caco-2 cells (for MjHKU4r-CoV-1 PsV), Caco2-*P.nat*-ACE2 cells (for HKU5-CoV-2 PsV) or Hela-*P.pip*-ACE2 cells (for NeoCoV and PDF-2180 PsVs) as target cells, which were plated in wells of a 96-well plate (10^4 cells per well) one day prior to infection. PsV was mixed with an equal volume of a peptide in indicated concentration for coincubation at 37°C for 30 min. Then, the mixture was transferred to the target cells. After 12 h, medium was refreshed for an additional 48 h culture. Finally, luciferase activity was analyzed by the Luciferase Assay System.

Circular dichroism spectroscopy and Tm value evaluation

EK1 (10 μM), MjHKU4r-HR1P (10 μM) and their mixtures were measured on a Jasco-815 circular dichroism spectrometer with scanning wavelength ranging between 200 and 260 nm. The $[\theta]_{222}$ value of "-33" (10^{-3} deg cm^2 $dmol^{-1}$) was taken to correspond to 100% α-helical. Thermal denaturation was detected at 222 nm with thermal gradient detection[4].

Native polyacrylamide gel electrophoresis (N-PAGE)

N-PAGE was conducted as described elsewhere. Briefly, EK1 (60 μM) in PBS was

incubated with HR1P peptide (50, 100, 200 μM), respectively, at 37°C for 30 min and then loaded on a 12% tris-glycine gel with tricine glycine running buffer (pH 8.6). Finally, gel staining was performed with Coomassie blue, and visualized on the FluorChem Imaging System.

Antiviral assays

As described previously, Caco-2 cells was used as target cell for authentic MjHKU4r-CoV-1 strain, which was plated in wells of a 48-well plate and cultured overnight. EK1 was serially diluted and incubated with MjHKU4r-CoV-1 strain (100 PFU) at 34°C for 30 min. The mixture was then transferred to target cell monolayers. After 1 h incubation, the mixture was removed. Target cells were washed with DMEM twice, and fresh DMEM containing 2% FBS and EK1 in indicated concentrations was added. At 72 h post-infection, viral RNA was extracted from culture supernatants for the quantification of viral genomic copies by RT-qPCR. Cells were fixed with 4% paraformaldehyde at room temperature for 40 min, and IF staining was performed, as described previously.

Expression and purification of fusion protein HR1-L6-EK1

The coding sequence of HR1 (residues 989–1,057) domain of MjHKU4r S2 subunit was tandem linked to EK1 though a 6-residue linker (L6: SGGRGG). The resulting sequence encoding the fused HR1-L6-EK1 protein was then cloned into a modified pET-32a vector containing a His_6-TRX tag upstream of the multiple cloning sites. The recombinant plasmid was expressed in Escherichia coli BL21. Cells were grown in lysogeny broth (LB) media supplemented with 50 μg/mL ampicillin at 37 °C after

inducing with 1 mM IPTG for 12 h at 16 °C overnight. Cells were harvested by centrifugation at 4500 g for 10 min at 4 °C. Then, cells were resuspended in buffer containing 25 mM Tris–HCl, pH8.0, and 200 mM NaCl and were lysed by high-pressure homogenizer. Fusion proteins were isolated by Ni-affinity chromatography, and the TRX tag was removed by TEV Protease (1:100 w/w) cleavage. HR1-L6-EK1 protein was concentrated and gel-filtered on a 10/300 Superdex 75 (GE Healthcare) column. Peak fractions containing HR1-L6-EK1 trimer were pooled and concentrated to 15 mg/ml through centrifugation.

Crystal structure determination

Crystals were obtained at 16°C for 5 days using the hanging drop vapor diffusion method by mixing equal volume of protein solution (MjHKU4r HR1-L6-EK1, 15 mg/mL) and reservoir solution (1M Sodium Citrate/Citric Acid, pH5.5, 20%(w/v) PEG 3000). Then crystals were flash-frozen and transferred to liquid nitrogen for data collection. On the in-house (Institute of Biophysics, Chinese Academy of Sciences) X-ray source MicroMax 007 generator (Rigaku, Japan) combined with Varimax HR optics (Rigaku, Japan), the crystals at 100 K were diffracted at a wavelength of 1.5418 Å. A native set of X-ray diffraction data was collected with the Satun944HG (Rigaku, Japan) with an exposure time of 15s per image and was indexed and processed using XDS. The space group of the collected dataset was C 1 2 1 for HR1-EK1 crystal. Molecular replacement was performed with PHENIX.phaser to solve the phasing problem, using crystal structure of SARS-CoV-2 HR1-HR2 (PDB entry 6LXT) as a search model. The final model was manually adjusted in COOT and refined with Phenix.refine. Data collection statistics and refinement statistics are

given in Table S1. Coordinates were deposited in the RCSB Protein Data Bank with entry code 8X5P.

References

1 Ma, C. B. *et al.* Multiple independent acquisitions of ACE2 usage in MERS-related coronaviruses. *Cell* **188**, 1693-1710.e1618 (2025).

2 Xia, S. *et al.* A pan-coronavirus fusion inhibitor targeting the HR1 domain of human coronavirus spike. *Sci Adv* **5**, eaav4580 (2019).

3 Xia, S. *et al.* Inhibition of SARS-CoV-2 (previously 2019-nCoV) infection by a highly potent pan-coronavirus fusion inhibitor targeting its spike protein that harbors a high capacity to mediate membrane fusion. *Cell Res* **30**, 343-355 (2020).

4 Yuan, C. *et al.* Heptad repeat 1-derived N peptide inhibitors improve broad-spectrum anti-HIV-1 activity. *Curr Res Microb Sci* **8**, 100364 (2025).

Supplementary Figures

a

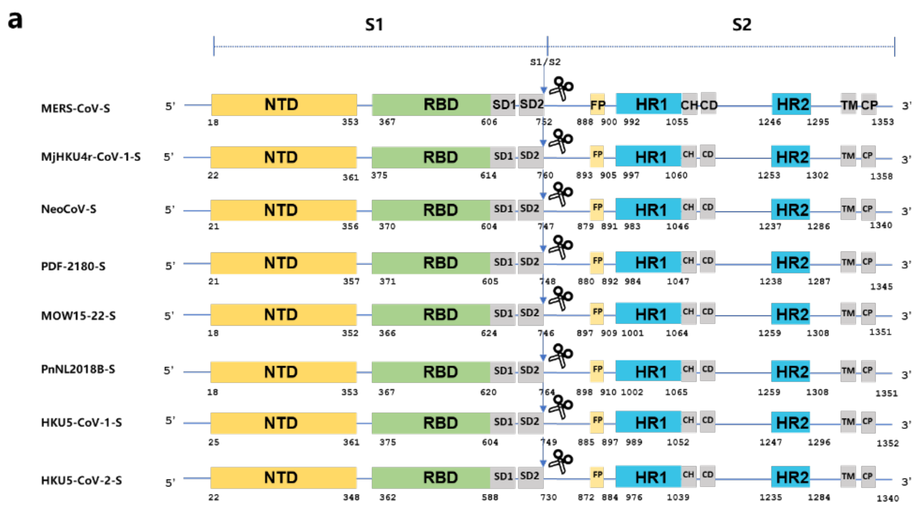

b

The identity of function domains in S protein						
MERSr-CoVs	S1 (%)	NTD (%)	RBD (%)	S2 (%)	HR1 (%)	HR2 (%)
NeoCoV	41.82	43.88	29.06	85.47	92.06	89.80
PDF-2180	43.27	46.87	31.62	87.61	92.06	91.84
MOW15-22	46.39	47.31	33.47	75.30	87.30	81.63
PnNL2018B	45.31	46.27	32.22	75.09	85.71	79.59
HKU5-CoV-1	55.86	55.22	51.53	71.38	85.71	67.35
HKU5-CoV-2	52.60	50.00	49.12	70.72	82.54	69.39
MjHKU4r-CoV-1	58.10	54.33	56.49	72.24	87.30	75.51
MERS-CoV	100.00	100.00	100.00	100.00	100.00	100.00

c

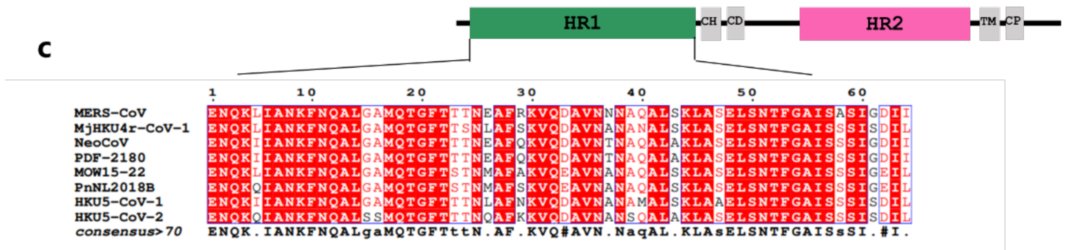

Supplementary Figure S1. Schematic representation of MERS-CoV or MERSr-CoV S protein.

a. The S protein of MERS-CoV and MERSr-CoVs includes NTD and RBD in the S1 subunit, and FP, HR1, and HR2 in the S2 subunit.

b. The identity of function domains in S protein of MERSr-CoVs, compared with those of MERS-CoV.

c. Sequence alignment of HR1 region among MERSr-CoVs and MERS-CoV. The same residues are shown in red background.

Supplementary Figure S2. The interactions between EK1 and MERSr-CoV

a

HR1-peptides	Sequence
MjHKU4r-CoV-1-HR1P	ANKFNQALGAMQTGFTTSNLAFSKVQDAVNANANALSKLASE
NeoCoV-HR1P	ANKFNQALGAMQTGFTTTNEAFQKVQDAVNTNAQALAKLASE
PDF-2180-HR1P	ANKFNQALGAMQTGFTTTNEAFQKVQDAVNTNAQALAKLASE
MOW15-22-HR1P	ANKFNQALGAMQTGFTSTNMAFAKVQEAVNANAQALSKLASE
PnNL2018B-HR1P	ANKFNQALGAMQTGFTSTNMAFSKVQEAVNANAQALSKLASE

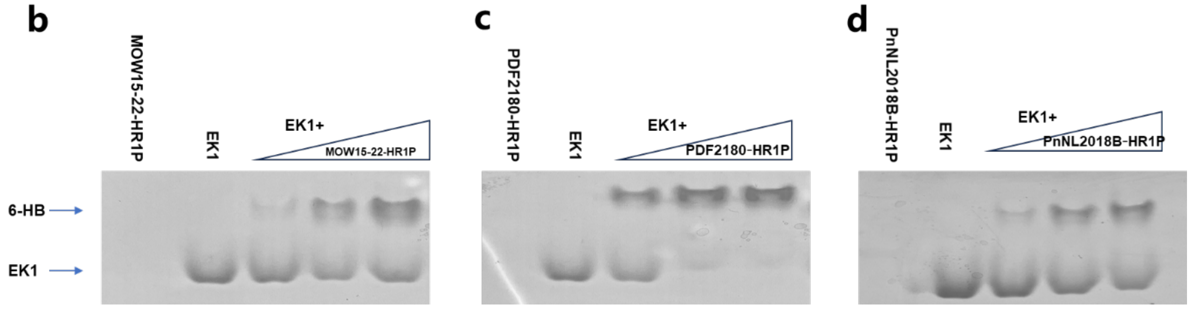

HR1Ps. a. The sequence of MERSr-CoV HR1Ps, PDF-2180-HR1P is same to NeoCoV-HR1P. **b-d**. Determination of the interactions between MOW15-22-HR1P (b) PDF-2180-HR1P (c) or PnNL-2180B-HR1P (d) with EK1 by N-PAGE.

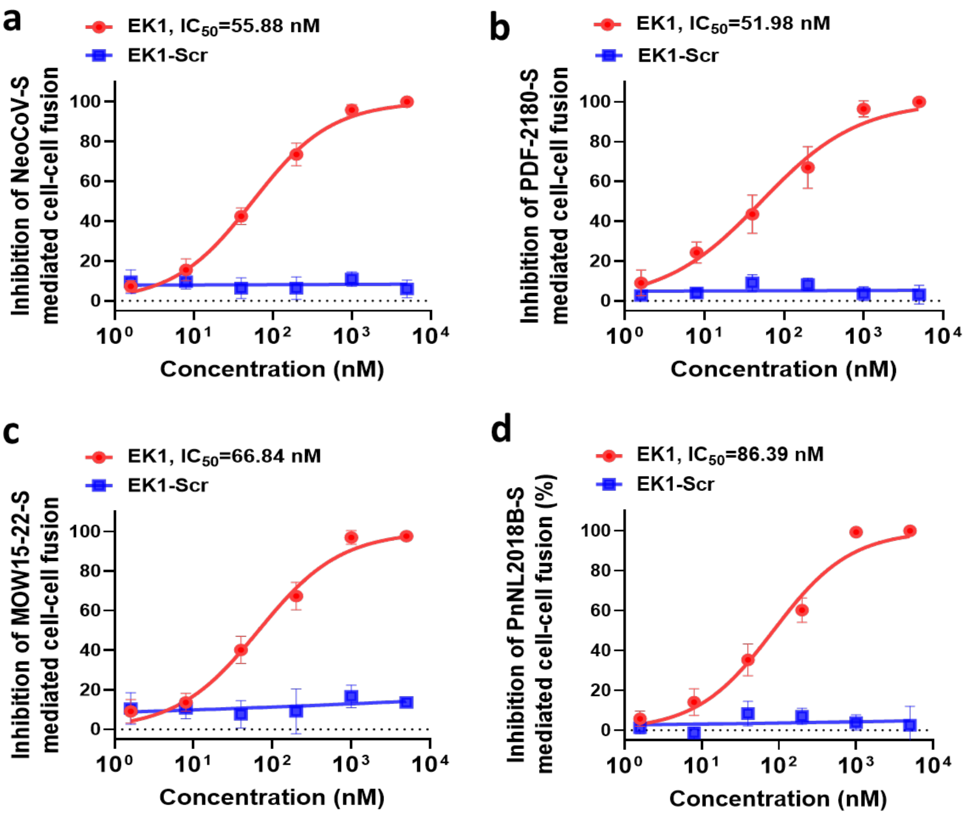

Supplementary Figure S3. The broad-spectrum efficacy of EK1 against MERSr-CoV-S-mediated membrane fusion process. Inhibitory activity of EK1 against cell-cell fusion mediated by NeoCoV-S (a), PDF-2180-S (b), MOW15-22-S (c) and PnNL2180B-S (d).

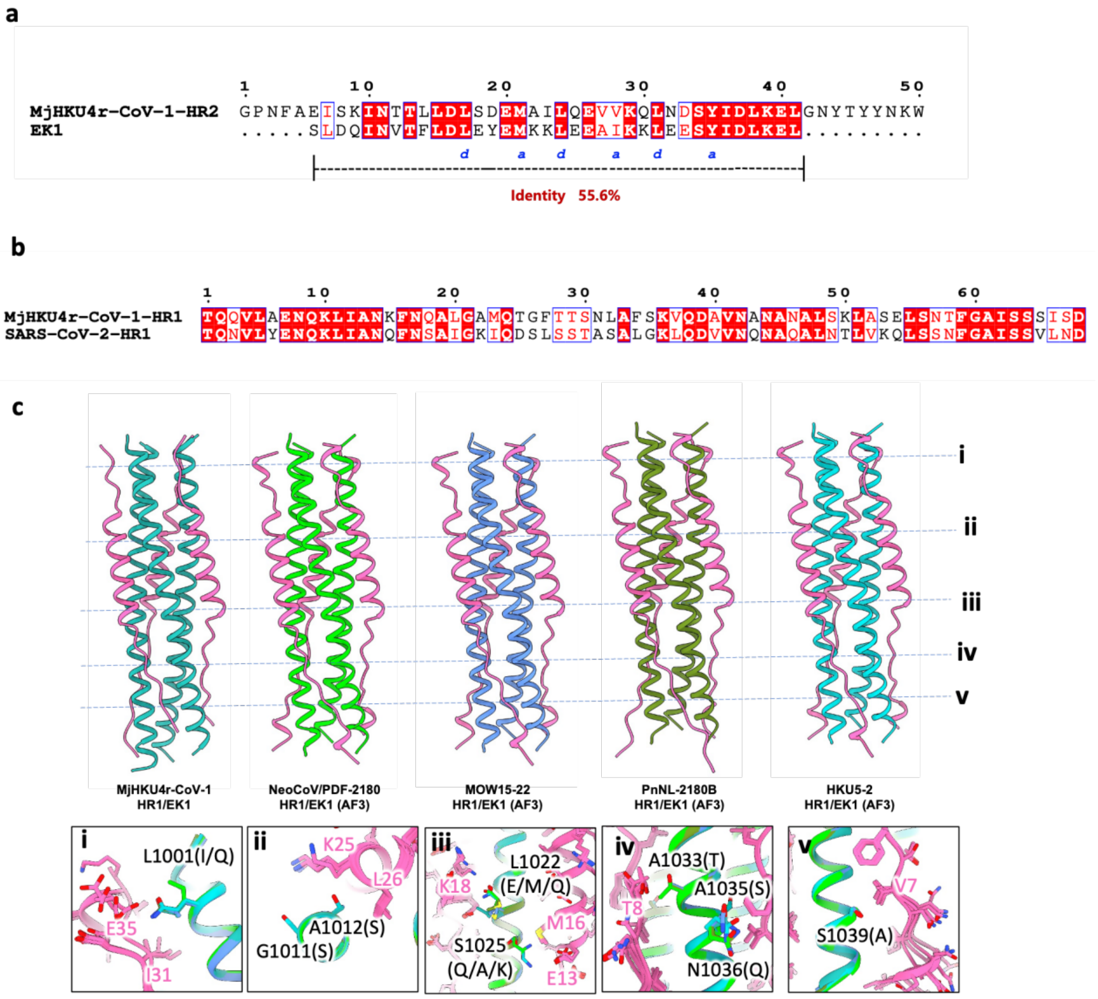

Supplementary Figure S4. Crystal structure of MjHKU4r-CoV HR1/EK1 complex.

Sequence alignment between EK1 and MjHKU4r-CoV-1-HR2 (a), and between MjHKU4r-CoV-1-HR1 and SARS-CoV-2-HR1 (b). Comparison of the structures in which EK1 binds to different MERSr-CoV HR1 domains (c). Except for MjHKU4r-CoV-1 (PDB entry 8X5P), the other structures are predicted using Alphafold3 (AF3). The HR1 domains are shown in different colors, and EK1 is displayed in pink. The local regions containing the mutation sites are enlarged for closer examination.

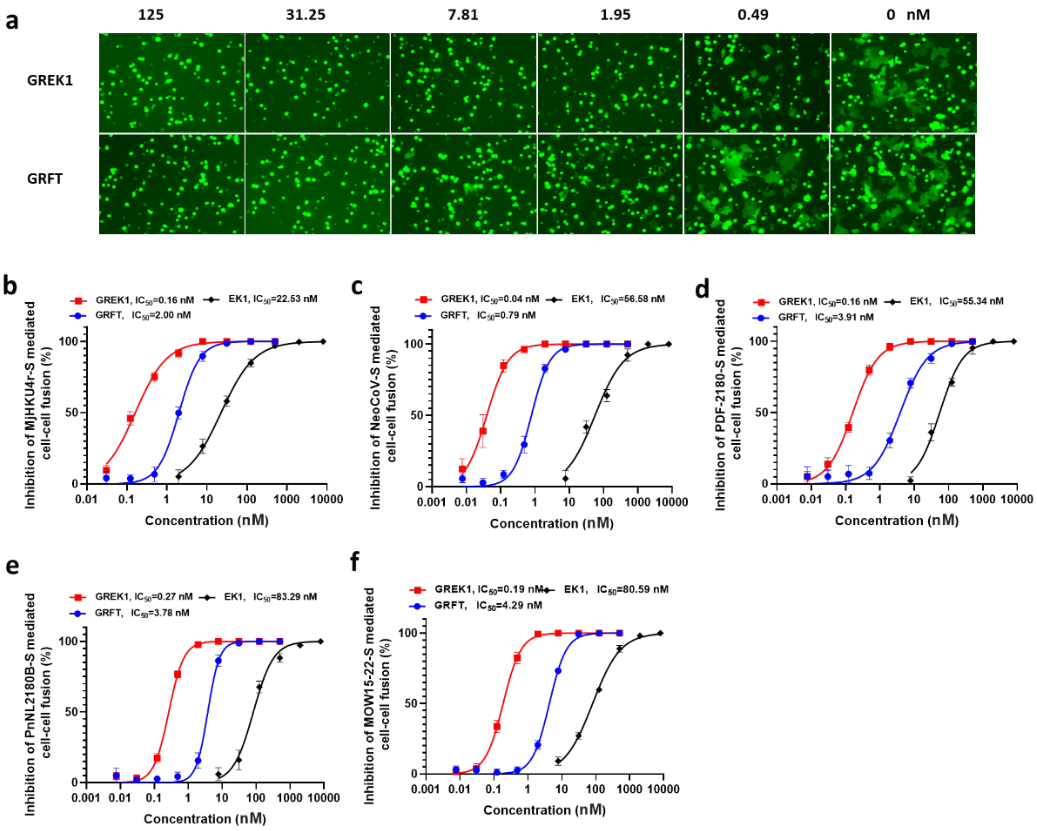

Supplementary Figure S5. Dual-targeting strategy exhibits extremely potent fusion-inhibitory activity against MERSr-CoVs.

a. Representative images of cell-cell fusion between effector cell expressing MjHKU4r-S and Caco2 cells after coculture for 2 h in the presence of GRFT or GREK1 with indicated concentration.

b-f. The broad-spectrum and potency of GREK1 against cell-cell fusion mediated by MjHKU4r-S (b), Neo-S (c), PDF-2180-S (d), PnNL2180B-S (e) and MOW15-22-S (f).

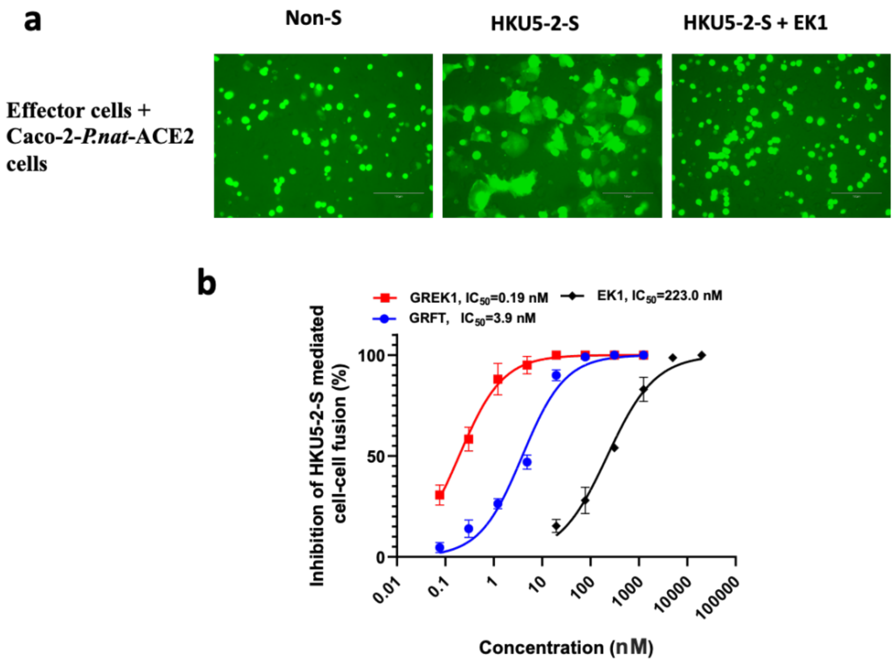

Supplementary Figure S6. GREK1 potently inhibited HKU5-CoV-2-S mediated membrane fusion and viral infection.

a. Representative images of EK1 (5 μM) blocking the HKU5-CoV-2-S mediated membrane fusion on Caco2-*P.nat*-ACE2 cells cells.

b. EK1, GRFT and GREK1 potently inhibited the HKU5-CoV-2-S mediated cell-cell fusion.

Supplementary Figure S7. EK1 and GREK1 potently inhibits propagation-

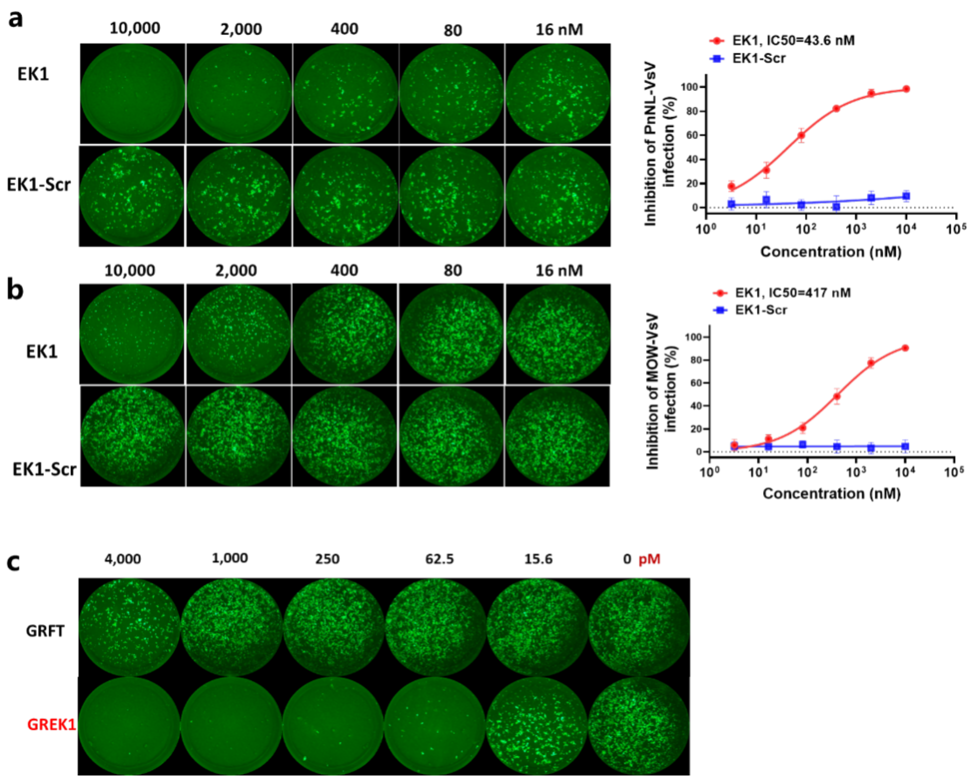

competent pcVSV-PnNL-2180B or pcVSV-MOW15-22 infection.

a. Representative images and quantification of propagation-competent pcVSV-PnNL-2180B infection on Caco2-*P.nat*-ACE2 cells for 48 h in the presence of EK1 or EK1-Scr with indicated concentration. Infected cells expressed GFP.

b. Representative images and quantification of propagation-competent pcVSV-MOW15-22 infection on Caco2-*P.nat*-ACE2 cells for 48 h with or without EK1 in indicated concentration. Infected cells expressed GFP.

c. Representative images of GRFT and GREK1 against pcVSV-MOW15-22.

Supplementary Table S1. Data collection and refinement statistics

	MjHKU4r-CoV-1 HR1-EK1 PDB entry 8X5P

**Data collection**	
Space group	C 1 2 1
Cell dimensions	
a, b, c (Å)	73.4, 42.3, 169.3
α, β, γ (°)	90, 98.2, 90
Wavelength (Å)	1.5418
Resolution (Å)	29.61-2.51(2.61-2.51)
R_{merge}	0.058(0.156)
Mean I/σ(I)	14.6(6.9)
Completeness (%)	94.5(95.3)
Redundancy	4.0(4.0)
**Refinement**	
Resolution (Å)	26.62-2.51 (2.6-2.51)
No. of reflections	16952
Reflections in test set	856
R_{work}/R_{free}	0.229/0.284
No. of atoms	
Protein	4297
Water & Ligands	143
r.m.s. deviations	
Bond lengths (Å)	0.007
Bond angles (°)	0.77
Ramachandran Outliers(%)	0.38
Average *B*-factor (Å^2)	30.04

†Highest resolution shell is shown in parenthesis.
